# It wasn’t me! Motor activation from irrelevant spatial information in the absence of a response

**DOI:** 10.3389/fnhum.2015.00539

**Published:** 2015-10-01

**Authors:** Carsten Bundt, Lara Bardi, Elger L. Abrahamse, Marcel Brass, Wim Notebaert

**Affiliations:** Department of Experimental Psychology, Ghent UniversityGhent, Belgium

**Keywords:** compatibility, grounded cognition, primary motor cortex, transcranial magnetic stimulation, motor evoked potential

## Abstract

Embodied cognition postulates that perceptual and motor processes serve higher-order cognitive faculties like language. A major challenge for embodied cognition concerns the grounding of abstract concepts. Here we zoom in on abstract *spatial* concepts and ask the question to what extent the sensorimotor system is involved in processing these. Most of the empirical support in favor of an embodied perspective on (abstract) spatial information has derived from so-called compatibility effects in which a task-irrelevant feature either facilitates (for compatible trials) or hinders (in incompatible trials) responding to the task-relevant feature. This type of effect has been interpreted in terms of (task-irrelevant) feature-induced response activation. The problem with such approach is that incompatible features generate an array of task-relevant and –irrelevant activations [e.g., in primary motor cortex (M1)], and lateral hemispheric interactions render it difficult to assign credit to the task-irrelevant feature *per se* in driving these activations. Here, we aim to obtain a cleaner indication of response activation on the basis of abstract spatial information. We employed transcranial magnetic stimulation (TMS) to probe response activation of effectors in response to semantic, task-irrelevant stimuli (i.e., the words left and right) that did not require an overt response. Results revealed larger motor evoked potentials (MEPs) for the right (left) index finger when the word *right* (*left*) was presented. Our findings provide support for the grounding of abstract spatial concepts in the sensorimotor system.

## Introduction

Embodied cognition interprets cognition as grounded in sensorimotor representations. This perspective on cognition has been supported, for example, by studies that demonstrated effector-specific activation of sensorimotor cortices during reading of action related words (Hauk and Pulvermüller, 2004; Hauk et al., 2004). Specifically, when the meaning of a verb is strongly linked to a specific action (e.g., “kick”, “pick”), mere reading of the verb evokes activation in cortical areas that are active during the actual execution of the respective action (Hauk and Pulvermüller, 2004). Furthermore, sensorimotor grounding has been found in action sentence comprehension (Aziz-Zadeh et al., 2006), and during auditory perception of action sentences (Buccino et al., 2005; Tettamanti et al., 2005).

While there exists ample support for sensorimotor grounding of concrete stimuli, there is an ongoing debate about how and to what extent abstract concepts are grounded in sensorimotor systems (for a review, see Pecher et al., 2011; Kiefer and Pulvermüller, 2012). For instance, the processing advantage (e.g., recall performance in memory tasks) for concrete over abstract concepts has been explained by proposing that concrete concepts are based on visual imaginary and verbal symbolic codes, while abstract concepts are only linked to the latter codes (Paivio, 1991). In order to relate abstract concepts to sensorimotor representations, frameworks were developed based on semantic processors that handle interpretation of concrete as well as abstract concepts (Mahon and Caramazza, 2008). Other frameworks emphasized the relevance of linguistic context (Schwanenflugel and Shoben, 1983), or focused on simulation of concrete situations that instantiate abstract concepts (Barsalou and Wiemer-Hastings, 2005). Thus, there exist diverse opinions about how abstract concepts are grounded in sensorimotor systems. Despite the ongoing controversy, understanding how (if at all) abstract concepts are represented in sensorimotor systems exemplify an important test case for the question whether concepts are embodied as a rule (e.g., Dove, 2015), and as such determines the reach of embodied cognition in general. Here, we zoom in on the question about whether abstract spatial concepts (“left” and “right”) are laid down in the sensorimotor system. Specifically, we investigate whether the processing of the words *left* and *right* is directly reflected in primary motor cortex (M1) activation. Previous research has delivered a number of indications that such M1 activation can be expected, though this conclusion has not yet been confirmed conclusively. Now, we will first outline the previous work that we build on.

Empirical evidence has shown that motor responses were modulated by implicit spatial stimulus features such as location, which may provide a first indication of an association between spatial stimulus information and spatially defined motor activation. The link between spatial stimulus information and motor responses has a long history in spatial compatibility research where responses to the task-relevant features are influenced by the processing of task-irrelevant spatial location of the stimulus (Lu and Proctor, 1995; Hommel, 2011). When the stimulus location feature is incompatible with the correct response side, reaction times (RTs) are longer and errors increase. Conversely, on compatible trials RT and error performance typically improves. Thus, incompatible stimulus-features can have a significant impact on goal-directed behavior. Interestingly, the performance decrease on incompatible Simon trials was shown to be accompanied by an (initial) ipsilateral activation of motor cortices (Valle-Inclán and Redondo, 1998; Vallesi et al., 2005). This could indicate that the task-irrelevant location feature initially triggers its corresponding motor activation. Similarly, a transcranial magnetic stimulation (TMS)—electromyography (EMG) study supports these findings by showing that stimulus location on incompatible trials in the Simon task is linked to heightened corticospinal excitability for the non-involved hand (van Campen et al., 2014). Thus, these studies suggest that there exists an association between (task-irrelevant) spatial stimulus information and spatially defined motor activation.

Furthermore, there is some indication that the semantic interpretation of spatially defined categories such as *above* or *below* interacts with the processing of location information. In a variant of the spatial Stroop task individuals are asked to respond to the location of a word that is compatible or incompatible with its meaning; for example, the word *above* printed above (compatible) or below (incompatible) a reference point (Seymour, 1973; O’Leary and Barber, 1993; Luo and Proctor, 2013). Responses to incompatible stimuli are typically slower than responses to compatible stimuli because the task-irrelevant word is processed which facilitates or interferes with responding to the relevant feature. This interaction indicates a link between semantics and stimulus location processing. More specifically, it suggests that both accessing stimulus semantics and the processing of stimulus location modulates motor activation and compete with each other (presumably) at the motor output level. One study using the spatial Stroop task in combination with the event-related optical signal (EROS) technique reported that stimulus semantics could generate activation at the level of the M1 (DeSoto et al., 2001), which suggests that spatial categories may be grounded in the sensorimotor system. In this study, a cue at the beginning of each trial determined which stimulus feature (i.e., semantics or location) was relevant on the current trial and individuals were asked to provide a response according to the relevant feature. However, DeSoto et al. (2001) did not distinguish between these two trial types; instead, they based their analysis on M1 activation during compatible and incompatible trials across the two tasks. Activation of M1 may have been based on both stimulus-driven response competition and response execution, which makes it impractical to investigate the isolated impact of single stimulus features (e.g., semantics) on M1 activation. Specifically, M1 activation may be confounded by competitive response execution processes that are due to the processing of two (potentially competing) stimulus features that both generate M1 activation.

In line with the findings from the spatial Stroop paradigm, other studies demonstrated that the processing of semantic, spatially defined categories could influence motoric components such as reaching and grasping kinematics (Gentilucci and Gangitano, 1998; Gentilucci et al., 2000; Glover and Dixon, 2002; Glover et al., 2004, 2005; Till et al., 2014). For instance, Glover and Dixon (2002) showed that the processing of the words *large* or *small* could modulate grip aperture early in the reaching movement. This effect was also found when words implicitly referred to large or small graspable objects (Glover et al., 2004). These studies suggest that semantic classifications could activate motor tendencies and translate to reaching and grasping kinematics. The neural analog of semantic classification was not investigated in these studies, and similarly to the studies mentioned above, results were contingent on interference effects (i.e., properties of the graspable object interfered with semantic classification) and response execution. Thus, the specific role of M1 during semantic classification remains unclear.

The reviewed studies show that: (i) implicit stimulus location—although task-irrelevant—changes motor activation; (ii) accessing semantic spatial information such as *above* may interact with motor activation that was generated by stimulus location; and (iii) processing abstract semantic stimuli such as *large* modulates motoric components like reaching and grasping kinematics. These studies all suggest a link between spatial information and motor activation and provide support for sensorimotor grounding of spatial information (location as well as more abstract semantic concepts). However, all of these studies made use of a compatibility paradigm where irrelevant information interacts with an overt response. Therefore, the observed effects are difficult to interpret as they might reflect complicated interactions between the processing of relevant and irrelevant information. Furthermore, in the studies that measured activation in motor areas of the brain, brain activation patterns may be confounded by stimulus-driven response competition resulting in overt response execution. More specifically, incompatible features generate an array of task-relevant and –irrelevant activations (e.g., in M1), and lateral hemispheric interactions (Chen, 2004) render it difficult to assign credit to the task-irrelevant feature* per se* in driving these activations. This is the reason why in these studies the isolated effect of single spatial stimulus features or single abstract spatial concepts on motor activation is impractical to examine. It remains unclear, therefore, to what extent the processing of abstract spatial concepts—like the words *left* or *right—*can generate spatially defined motor activation when response execution and stimulus-driven response competition is prevented.

As noted above, the present study sought to investigate whether the processing of (abstract) semantic concepts is reflected in M1 activation, even when no overt response is required. In our set-up, participants are passively watching the words *left* or *right* presented centrally on the screen, while we measure whether this induces corresponding motor activation. Importantly, from behavioral studies we know that participants need to be engaged in a left-right discrimination task before we can observe activation on the basis of horizontal spatial information (Hommel, 1996; Ansorge and Wühr, 2004, 2009; Wühr and Ansorge, 2007; Zhao et al., 2010). Therefore, we implemented trials where participants had to respond with a left or right keypress to colored circles. These trials were implemented so that a left-right discrimination was part of the overall task set, even though we measured motor activation on trials were no response was required. On word trials, spatial words LINKS (Dutch for left) or RECHTS (Dutch for right) or non-words (XXXXX) were presented and participants were instructed to ignore these irrelevant stimuli. During these trials, TMS was applied to assess corticospinal excitability and motor evoked potentials (MEPs) were recorded from the left and right first dorsal interosseus (FDI). It was predicted that the respective FDI would be more activated by a compatible (e.g., right FDI and RECHTS) compared to an incompatible word (e.g., right FDI and LINKS), extending previous findings of the effect of task-irrelevant information on cognition.

## Materials and Methods

### Participants

Twenty two healthy, Dutch native speakers took part in the current study (20 female; mean age: 21.19 ± SD: 1.83) and were paid for their participation (35€). All participants gave written informed consent according to the declaration of Helsinki, had normal or corrected-to-normal vision and were prescreened for psychological, neurological and other factors that could interfere with a safe application of TMS (Rossi et al., 2009). Four participants were excluded from the final sample; two participants due to technical failure and two more because of an insufficient number of word (i.e., TMS) trials (see “Data Analysis” Section below). The study was approved by the Medical Ethical Review Board of the Ghent University Hospital.

### TMS Stimulation and EMG Recordings

EMG was obtained from the left and right FDI muscle, which is relevant for abducting the index finger away from the middle finger. EMG activity was recorded using the ActiveTwo system^1^. Sintered 11 × 17 mm active Ag-AgCl electrodes were placed over the right and left FDI, and reference electrodes were placed over the metacarpophalangeal joints, respectively. Furthermore, the ground-electrode was mounted onto the back of the right hand close to the wrist joint. The EMG signal was amplified (internal gain scaling) and digitized at 2048 Hz. Furthermore, a high-pass filter of 3 Hz was applied. For further offline analyses, resultant data was stored on a separate personal computer. A biphasic stimulator (Rapid2; The Magstim Company Ltd.) and a 70 mm figure of eight coil were used to deliver TMS pulses (for implications of TMS stimulation see Bestmann and Duque, 2015; Bestmann and Krakauer, 2015). The coil was held tangentially over the left (or right) hand motor area. The coil handle pointed backward and built an angle of 45° with the sagittal plane and was held by a mechanical arm during the experiment. The scalp location of TMS stimulation was dependent on the position at which the most reliable MEP was obtained. For each hemisphere, the intensity that evoked MEPs larger than 50 μV in 50% of the cases was defined as the resting motor threshold (rMT; Rossini et al., 1994) and determined the eventual TMS stimulation intensity for each subject and hemisphere. During the experiment, the stimulation intensity was set at 120% of the rMT (left M1 rMT: 54.94%; right M1 rMT: 54.16%). On average, the intensity was 64.18% (range 49–80%) of the maximal stimulator output. Subjects were outfitted with a swimming cap on which the location of TMS stimulation was highlighted. Using this method, the experimenter was able to continuously monitor the location of TMS stimulation.

### Stimuli and Procedure

Participants were seated in a comfortable armchair in a darkened and noise-shielded room. Participants were asked to put the tips of each index finger between two buttons (between F4 key and F5 key, and between F8 key and F9 key respectively) on a reversed standard QWERTY keyboard (for a similar procedure, see Klein et al., 2012, 2014). Furthermore, participants were instructed to provide a bimanual choice after the presentation of a relevant stimulus (specified further below), by performing an abduction movement with either the left or right index finger away from the middle-finger and towards a medial response button (F5 key and F8 key) to eventually execute a key press.

Experimental stimulus presentation was carried out on a 17-inch computer monitor (1024 × 768 pixels) using Presentation^®^ software (Version 16.3^2^) Half of all trials (*N* = 384) were color (i.e., non-TMS) trials, whereas the other half were word (i.e., TMS) trials.

**Figure 1:**
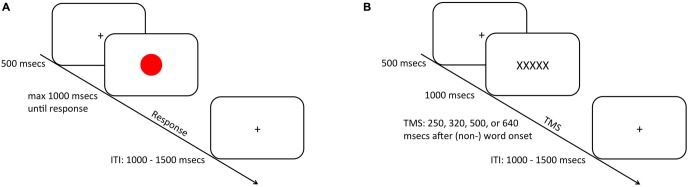
**Schematic representation of the trial procedure.** During half of the trials **(A)**, subjects were required to respond via a bimanual key press to the ink-color of a centrally presented circle that was presented for maximally 1000 ms depending on the speed of participant’s response. On the other half of the trials **(B)**, a (non-) spatial word was presented upon which the subjects did not provide any overt response. After one of four intervals 250, 320 or 500 (640 ms) a transcranial magnetic stimulation (TMS) pulse was applied over the primary motor cortex (M1) to probe M1 excitability. Trials were separated by an inter-trial-interval (ITI) that was jittered between 1000 and 1500 ms.

During color trials (i.e., non-TMS trials; Figure 1A) a presentation cross was presented for 500 ms. after which a red or a green circle (height and width: 1.7°) was presented centrally on the screen for maximally 1000 ms, upon which the participant had to provide a response according to the color of the stimulus. If the participant did not respond within the 1000 ms stimulus presentation window, a “too late” screen was presented for 1000 ms. On word trials (Figure 1B) the presentation of a fixation cross for 500 ms was followed either by a word inheriting spatial semantics (i.e., RECHTS; LINKS; Dutch for right and left respectively) or by a nonspatial control-word (i.e., XXXXX) (height: 0.7°; width: maximally 3.8°) displayed for 1000 ms. A TMS pulse was delivered after one of four stimulus-pulse intervals (250, 320, 500, or ms; c.f. Catmur et al., 2007). This resulted in 16 TMS pulses that were applied per hemisphere, condition, and timing (see “Data Analysis” Section). Crucially, participants were instructed not to provide any response during word trials. Individual trials were separated by a jittered inter-trial-interval (ITI) of 1000–1500 ms.

In total, participants needed to complete six blocks of 128 pseudo-randomized trials, respectively. Each block was separated by a 1 min break. After three blocks, the stimulated hemisphere was changed. The order of hemisphere stimulation was counterbalanced across participants. In total, the experiment took about 1.5 h.

### Data Analysis

Peak-to-peak amplitude of the MEP was calculated for each trial. EMG epochs starting 500 ms before and ending 500 ms after the actual event (i.e., the TMS pulse) were extracted from the recorded data. Trials were checked for background EMG activity during a time window of 500 ms preceding the TMS pulse. The trial was rejected if background EMG activity was found during this window. Using MATLAB software, peak-to-peak MEP amplitude of each trial was calculated for the 20–40 ms window following a TMS pulse (i.e., this is the typical time range at which a MEP occurs). Subsequently, the total number of trials that survived preprocessing was calculated for each subject. The (population) mean number of trials was 13.79 (SD ± 3.24) averaged across all conditions and subjects. Subjects were removed from further analysis when the mean amount of trials across all conditions fell two standard deviations or more below the average number of trials across all subjects and conditions (*N* = 2 individuals). Thus, the final sample on which statistical analyses were performed consisted of 18 individuals. On average, this procedure resulted in 14.37 (SD ± 2.46) trials per condition (i.e., stimulated hemisphere, compatibility and TMS timing). Moreover, due to the highly variable nature of MEPs in participants and to avoid MEP amplitude variability affecting subsequent analyses unevenly *Z*-scores normalization was performed (Burle et al., 2002; van den Wildenberg et al., 2010). First, the mean and the standard deviation were calculated for all valid trials (i.e., trial population mean) per participant. Thereafter, *Z*-scores were computed by subtracting the trial population mean from the individual trial MEP amplitude and dividing it by the trial population standard deviation of the respective subject. *Z*-scores were then averaged per condition and subject. Resulting MEP data were submitted to a 2 × 3 × 4 repeated measures ANOVA with hemisphere (left, right) × compatibility (compatible, incompatible, neutral) × timing (250, 320, 500, 640 ms) as within-subject factors. Potential effects were further investigated using paired-sample *t*-tests. All statistical tests were carried out using SPSS (Version 22.0. Armonk, NY, USA: IBM Corp.). The statistical significance threshold was set to *p* = 0.05. Whenever necessary, the Greenhouse-Geisser epsilon correction as well as the Bonferroni correction were applied.

## Results

Color trials. The mean RT and the mean proportion of correct responses were 591.04 ms (SD ± 39.92) and 98.13% (SD ± 0.016) respectively.

**Figure 2:**
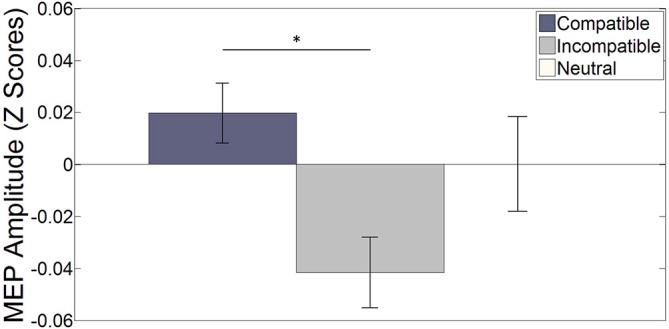
**The bar plot shows the effect of (non-) spatial words on the (in-) compatible effector averaged over both hemispheres and all four stimulation intervals.** Error bars depict the standard error of the mean. *p < 0.05. On average, MEP amplitudes were larger for compatible stimuli compared to incompatible stimuli (*t*_(17)_ = 3.101, *p* = 0.006). The difference between compatible and neutral and incompatible and neutral stimuli did not reach significance (*t*_(17)_ = 0.825, *p* = 0.421) and (*t*_(17)_ = −1.606, *p* = 0.127) respectively.

**Figure 3:**
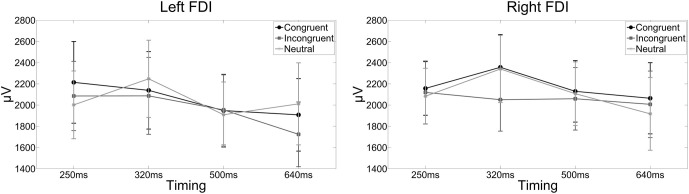
**The line graphs show the raw MEP amplitudes for each condition and FDI for illustrative purposes.** Error bars indicate standard errors of the mean. Actual statistical tests were run on the *Z* scores only. The left line graph shows the raw MEP amplitudes in the left FDI when a compatible, incompatible or neutral word was presented and corticospinal excitability was assessed 250, 320, 500, or 640 ms after word onset. The right line graph shows the raw MEP amplitudes for the right FDI when a compatible, incompatible or neutral word was presented and corticospinal excitability was assessed 250, 320, 500, or 640 ms after word onset.

Word trials. Figure 2 shows the normalized Z-score MEP amplitudes averaged over hemisphere and stimulation interval for each specific stimulus during word trials (see Figure 3 for raw MEPs). Results indicate a main effect of compatibility (*F*_(2, 34)_ = 3.613, *p* = 0.038, η^2^ = 0.175). A *paired-sample t-test* indicates a significant difference between compatible and incompatible stimuli (*t*_(17)_ = 3.101, *p* = 0.006, *r*^2^ = 0.361). This illustrates increased MEPs for the left (right) index finger when the word LEFT (RIGHT) is presented compared to when the word RIGHT (LEFT) is presented. The difference between compatible trials and neutral, and incompatible trials and neutral trials did not reach significance, (*t*_(17)_ = 0.825, *p* = 0.421) and (*t*_(17)_ = −1.606, *p* = 0.127), respectively.

Furthermore, a main effect of stimulation interval was observed (*F*_(1.758,29.889)_ = 5.157, *p* = 0.015, η^2^ = 0.233), indicating a reverse relationship between MEP amplitude and stimulation interval. No effect of hemisphere, however, was observed (*F*_(1, 17)_ = 0.488, *p* = 0.494, η^2^ = 0.048), and none of the interactions reached significance (*p* > 0.05).

## Discussion

There exists ample evidence for sensorimotor grounding of concrete action words and sentences (Hauk and Pulvermüller, 2004; Hauk et al., 2004; Buccino et al., 2005; Tettamanti et al., 2005; Aziz-Zadeh et al., 2006), for the influence of higher-order semantic classification on motoric components such as reaching and grasping kinematics (Gentilucci and Gangitano, 1998; Gentilucci et al., 2000; Glover and Dixon, 2002; Glover et al., 2004, 2005; Till et al., 2014), and for an interaction between location information and processing of spatial semantic categories (Seymour, 1973; O’Leary and Barber, 1993; Luo and Proctor, 2013). The current results add to these findings by providing the strongest evidence so far that the processing of the abstract, spatial concepts ‘left’ and ‘right’ is associated with activation (i.e., M1 excitability) in sensorimotor systems—when critically no overt response was required. To our knowledge, this is the first time that motor activation on the basis of abstract spatial information has been demonstrated at the level of M1 when response execution and response competition driven by multiple and potentially incompatible stimulus-features is prevented. Our results strengthen the weakest empirical link of the embodied cognition perspective by supporting the notion that even abstract spatial concepts are grounded in sensorimotor systems. According to dis-embodied views on cognition, abstract spatial concepts should not activate the sensorimotor system when no further response is required, and this is clearly not what we observed here.

Showing M1 activation based on the processing of the words *left* and *right* is an important step towards a successful defense of the embodied perspective. Yet, one may argue that the activation is a non-critical side-effect of this processing and thus does not entail a true indication of grounding. Pulvermüller (2005) describes three criteria for demonstrating grounded cognition. The first criterion is speed. The observed effects should be fast. In the current study, TMS stimulation was executed as early as 250 (to 640) ms after word onset, and an effect of compatibility on hemisphere-specific motor activation was observed independent of TMS timing. This suggests a *fast* modulation of corticospinal excitability by abstract, spatial and semantic information and thus confirms the first criterion by Pulvermüller (2005). However, whether comparable effects on corticospinal excitability could be observed when TMS stimulation was implemented at earlier intervals needs yet to be determined.

Second, the effect should be somatotopic. Translated to our context, this criterion entails that a lateral, hemisphere-specific effect should be observed in the sense that the word *left (right)* results in right(left) M1 motor activation. This criterion was confirmed in current study. Specifically, the results indicate that the perception and semantic interpretation of spatial information can lead to selective activation of M1. Larger stimulus-induced corticospinal excitability has been obtained on compatible trials for the corresponding M1, while corticospinal excitability was significantly smaller when the semantics of the spatial stimulus did not correspond with the effector location (i.e., hemisphere-specific motor activation). Thus, the somatotopic criterion by Pulvermüller (2005) is also met.

Third, the activation should be automatic. In the current context this demands that focused attention towards the semantic feature of the stimulus is not required to execute the task and thus to generate sensorimotor cortex activation. In our experiment, the semantic stimulus does not hold any task-relevant feature to respond to, and thus no feature that requires focused attention. Indeed, already its mere surface features (shape, color, et cetera) are fully informative about the fact that on this trial no response is required. This satisfies the third criterion by Pulvermüller (2005). One may object that in our design, half of the trials required a left-right discrimination on the basis of the color of centrally presented circles, and this may have resulted in systematic pre-stimulus preparation of both response alternatives. This is perhaps true, but our main point is that we observed an asymmetrical increase of activation post-stimulus onset for one of two response alternatives based on the spatial word, which is difficult to explain based on (symmetrical) pre-stimulus preparatory mechanisms only. Overall we believe that the current results can be taken to indicate grounding of abstract spatial concepts in the sensorimotor system.

Furthermore, results show that the amplitude of MEPs decreases with increasing TMS latency. In general, it has been observed that response inhibition is associated with a decrease of MEP amplitude (van den Wildenberg et al., 2010). Moreover, this decrease of amplitude is contingent on the latency of the TMS pulse (Yamanaka et al., 2002). In line with these studies, we interpret our finding of a main effect of TMS latency as depicting response inhibition after the individual realized that he/she does not have to respond on the current trial. Consequently, corticospinal excitability and MEP amplitude decreases. Importantly, this decrease is observed irrespective of the stimulus. The selective motor excitability does not depend on time, in the sense that there is no interaction between the factors timing and compatibility.

The intermixing of color trials served a clear purpose in our study. On the basis of previous work (Hommel, 1996; Ansorge and Wühr, 2004, 2009; Wühr and Ansorge, 2007; Zhao et al., 2010) we predicted that without those trials, no motor activation would have been observed because this requires response discrimination in working memory. For instance, in a series of experiments, Ansorge and Wühr (2009) observed a Simon effect in a go/no-go task (requiring uni-manual detection responses in go-trials) only when it was preceded by a choice-response task and when both tasks shared stimulus-response mappings. Conversely, before the choice-response task there was no reliable Simon effect in the go/no-go task. The Simon effect in the former case was assigned to a transfer of the required response discrimination in working memory from the choice-response to the go/no-go task. Based on this type of finding, we decided to include the color trials to induce response discrimination in our participants. However, our design provides a strong paradigm to further test the notion of response discrimination. It would certainly be interesting to examine whether the processing of abstract spatial concepts modulates hemisphere-specific corticospinal excitability without the implementation of bimanual responses that need to be discriminated along a spatial axis. For instance, what would we observe if we delete the color-trials all together, and just let participants passively watch the spatial concepts be presented? More intermediate steps to examine the (unconditional) nature of embodiment of abstract spatial concepts may also be interesting. For example, one may ask individuals to respond to the color of stimuli via spatially defined, verbal responses (e.g., green circle, say ‘right’). In this scenario, the individual effectively only distinguishes between spatial categories vocally and need not rely on bimanual right/left motor discriminations. If in this scenario similar MEP modulation is observed, this would hint at the possibility that a semantic (instead of a motoric) discrimination between (response) location alternatives may already be sufficient—broadening the perspective to a cognitive discrimination account. Hence, the current design has great promise for future exploration of issues related to automaticity. One may also argue that in the current study the color trials are only indirectly linked to spatial response discrimination, because color stimuli did not inherently contain spatial (i.e., lateralized) properties. It could therefore also be interesting to examine the impact of spatial stimuli without spatial responses on the automatic motor activation as we observed it. More specifically, one could introduce lateralized stimuli and ask individuals to respond verbally in a non-lateralized fashion (e.g., left circle, say boo) while intermixing these trials with word trials. In this setup and according to the response-discrimination account, we would assume not to find the effects observed in the current study, because responses do not need to be distinguished along a spatial axis anymore.

Based on the three criteria pinpointed by Pulvermüller (2005), the current study fits the notion of grounded representation of abstract spatial concepts. Several cognitive frameworks have been introduced to substantiate the mechanisms underlying such grounded cognition. For example, Barsalou and Wiemer-Hastings (2005) proposed that abstract concepts are instantiated by the simulation of concrete situations to which the abstract concept applies. Thus, abstract concepts could (partly) be grounded in sensorimotor systems because they evoke simulation of concrete situations. However, the simulation of concrete vs. abstract stimuli differs in terms of focal content. The content of abstract concepts is less focal because there are numerous concrete situations upon which the stimulations could be based. The broader representation of abstract concepts may therefore be associated with distributed and more complex representations at the brain level (Pexman et al., 2007) and may vary depending on contextual and situational constraints (Hoenig et al., 2008). This framework of instantiating abstract concepts via simulation is coherent with studies that have shown that individuals are better in comprehending abstract material, when a linguistic context was provided compared to when the abstract material was presented in isolation (Schwanenflugel and Shoben, 1983). In current study, the concrete context may serve as anchor on which simulation is based. Thus, the implementation of right/left categories during color trials may provide the specific context where individuals could base their simulations upon.

Alternatively, the *grounding-by-interaction* framework Mahon and Caramazza (2008) suggests that sensory and motor information is important to provide an enriched context for conceptual processing. Instantiating abstract concepts is linked to the reactivation of sensory and motor information and would thereby ground conceptual representations in the sensorimotor system. In contrast to Barsalou and Wiemer-Hastings (2005) who are not specific about the consequences if individuals are unable to simulate concrete situations (e.g., apraxic patients), Mahon and Caramazza (2008) proposed that when conceptual processing would lack motor and sensory information, concepts would severely be impoverished but they would continue to exist in this impoverished form. Thus, although conceptual representations can be generalized and are flexible in the sense that they can be applied to numerous concrete situations, information from sensorimotor (i.e., concrete) systems may provide a richer environment to better process conceptual representations.

Present results could be explained in line with the assumption that abstract concepts may benefit from simulating concrete situations. During half of the trials, individuals needed to discriminate between response alternatives and therefore, needed to distinguish between spatial categories (i.e., left and right). During word trials, this discrimination may have served as concrete situation on which simulations of abstract spatial words (*left* and *right*) was based upon. Thus, without color trials, simulating a concrete situation in which the spatial categories left and right are of relevance and are linked to sensorimotor experiences may be more difficult.

One limitation of current study may be the choice for the abstract spatial concepts “left” and “right”. These concepts are surely abstract and spatial in themselves because they are not, for instance, spatially constraint or purely physically defined (Barsalou and Wiemer-Hastings, 2005). However, the implementation of these concepts is often required in daily life. For instance, when a person looks for a specific product in the supermarket and is told that the product is *to the left*, the individual needs to implement the concept *left* (*right*) in order to find the product she is looking for. Correspondingly, the frequency with which this spatial concept is motorically implemented in daily life may strengthen the concept-sensorimotor activation link and may shift abstract spatial concepts towards a more concrete interpretation with accompanying activation in sensorimotor brain regions. Alternatively, this spatial concept may easier be implemented than other abstract concepts (e.g., truth, freedom) due to the sheer number of available situations where this concept is implemented on a daily basis. Thus, spatial abstract information such as *left* (*right*) may have a processing advantage over other abstract concepts (e.g., freedom, truth) and may be accompanied by improved or heightened sensorimotor activation.

In conclusion, our results suggest that incidental processing of abstract spatial concepts is reflected in effector-specific M1 activation even though no response is required. These findings are coherent with the view that abstract concepts may be instantiated by simulating concrete situations and add to the discussion of sensorimotor grounding of abstract concepts.

## Conflict of Interest Statement

The authors declare that the research was conducted in the absence of any commercial or financial relationships that could be construed as a potential conflict of interest.

## Footnotes

^1^www.biosemi.com

^2^www.neurobs.com
